# Towards a dynamic com­pression facility at the ESRF

**DOI:** 10.1107/S1600577521011632

**Published:** 2022-01-01

**Authors:** Nicolas Sévelin-Radiguet, Raffaella Torchio, Gilles Berruyer, Hervé Gonzalez, Sébastien Pasternak, Florian Perrin, Florent Occelli, Charles Pépin, Arnaud Sollier, Dominik Kraus, Anja Schuster, Katja Voigt, Min Zhang, Alexis Amouretti, Antoine Boury, Guillaume Fiquet, François Guyot, Marion Harmand, Marcello Borri, Janet Groves, William Helsby, Stéphane Branly, James Norby, Sakura Pascarelli, Olivier Mathon

**Affiliations:** a European Synchrotron Radiation Facility, 71 Avenue des Martyrs, CS 40220, 38043 Grenoble, France; b CEA, DAM, DIF, 91297 Arpajon Cedex, France; c Université Paris-Saclay, CEA, Laboratoire Matière en Conditions Extrêmes, 91680 Bruyères-le-Châtel, France; d Helmholtz-Zentrum Dresden-Rossendorf, Bautzner Landstrasse 400, 01328 Dresden, Germany; e Institut für Physik, Universität Rostock, Albert-Einstein-Strasse 23–24, 18059 Rostock, Germany; f Technische Universität Dresden, 01069 Dresden, Germany; g Institutes of Physical Science and Information Technology, Anhui University, 230601 Hefei, People’s Republic of China; h Institut de Minéralogie, de Physique des Matériaux et de Cosmochimie, UMR 7590 – Sorbonne Université/CNRS/MNHN/IRD, 75252 Paris, France; i STFC, Daresbury Laboratory, Warrington, United Kingdom; j Amplitude Technologies, 2–4 Rue du Bois Chaland, CE 2926, 91029 Évry, France

**Keywords:** dynamic com­pression, laser shock, time-resolved XAS

## Abstract

Results of the 2018 commissioning and experimental campaigns of the new High Power Laser Facility on the Energy-dispersive X-ray Absorption Spectroscopy (ED-XAS) beamline ID24 at the ESRF are presented. The front-end of the future laser, delivering 15 J in 10 ns, was interfaced to the beamline. Laser-driven dynamic com­pression experiments were performed on iron oxides, iron alloys and bismuth probed by online time-resolved XAS.

## Abstract 2

The results of the 2018 commissioning and experimental campaigns of the new High Power Laser Facility on the Energy-dispersive X-ray Absorption Spectroscopy (ED-XAS) beamline ID24 at the ESRF are presented.

## 1. Introduction

Dynamic com­pression induced by powerful lasers offers a route to extreme conditions of pressure and temperature that go beyond what is achievable today by static com­pression with the laser-heated diamond anvil cell. The capability of reproducing and probing such extreme conditions in matter is relevant for planetary and exoplanetary science (Remington *et al.*, 2006*b* ▸; Kraus *et al.*, 2017 ▸; Noack & Lasbleis, 2020 ▸), fundamental studies of condensed matter (Grochala *et al.*, 2007 ▸), industrial applications such as new materials synthesis (He *et al.*, 2001 ▸) and energy science for Inertial Confinement Fusion (ICF) (Dittrich *et al.*, 1999 ▸). (Exo)planetary science is witnessing a revolution with the discovery of thousands of extra­solar planets orbiting nearby stars (Batalha, 2014 ▸). Characterizing such planetary bodies requires knowledge of the physical properties (structural and electronic) of the key constituents at pressures of multi-Mbar and temperatures of a few thousands of Kelvin. In the interior of the Earth and planets, matter can be found in the Warm Dense Matter (WDM) state, somewhere between the solid and plasma state. Such an intriguing regime (Riley, 2017 ▸) is still challenging to describe by theoretical models, as conventional approximations, that apply to solids or ideal plasmas, break down and since experimental data are scarce. WDM states are also encountered in ICF during laser-induced target com­pression.

Under more moderate conditions, dynamic com­pression can be used to study the behaviour of materials under high strain rates (Rusty & George, 2012 ▸). Phase diagrams obtained under dynamic com­pression can differ greatly from the static case and there is a need to understand the strain rate and temperature dependence of kinetics, phase nucleation, metastability, elasticity and plasticity effects (Mulliken & Boyce, 2006 ▸; Gleason *et al.*, 2015 ▸; Briggs *et al.*, 2019 ▸; Gorman *et al.*, 2018 ▸; Pépin *et al.*, 2019 ▸). This area of research also encom­passes industrial processes such as laser machining or the synthesis of new materials. Under dynamic conditions, new chemistry can occur (*i.e.* Celliers *et al.*, 2018 ▸; Millot *et al.*, 2019 ▸; Brygoo *et al.*, 2021 ▸) or new phases can be formed (Zhang *et al.*, 2020 ▸).

Historically, such science has been predominantly developed at large laser facilities, such as the OMEGA laser (Hoose, 1977 ▸; Boehly *et al.*, 1997 ▸) and the National Ignition Facility (NIF) (Miller *et al.*, 2004 ▸) in the USA or the LULI2000 (Koenig *et al.*, 2006 ▸) in France, where most extreme states can be induced up to the TPa range. Secondary X-ray probes may be produced *in situ* at the expense of a large part of the laser energy to drive plasma excitation or capsule implosion (Harmand *et al.*, 2009 ▸; Benuzzi-Mounaix *et al.*, 2011 ▸; Ping *et al.*, 2013 ▸; Denoeud *et al.*, 2014 ▸; Coppari *et al.*, 2017 ▸; Krygier *et al.*, 2018 ▸). A notable example is the development of X-ray absorption spectroscopy (XAS) measurements at NIF and OMEGA, where good-quality EXAFS (extended X-ray absorption fine structure) could be obtained under ambient conditions (Coppari *et al.*, 2017 ▸; Krygier *et al.*, 2018 ▸) and ramp-com­pressed Fe (Ping *et al.*, 2013 ▸). Moreover, recently, much effort has been devoted to the improvement of the energy resolution so as to be able to observe XANES features (Chin, 2020 ▸). However, user access to these facilities remains very limited due to the low shot rate.

In recent years, a new strategy has emerged: coupling more com­pact lasers to brilliant X-ray sources such as synchrotrons or XFELs to ensure high-quality X-ray measurements (Glenzer *et al.*, 2016 ▸; Wang *et al.*, 2019 ▸; Inubushi *et al.*, 2020 ▸), thus exploiting the long-standing expertise of the synchrotron community.

In this context, the ESRF has developed the opportunity of coupling dynamic com­pression experiments to time-resolved single-pulse XAS at beamline ID24. Laser-shocked Fe (Torchio *et al.*, 2016 ▸) and Ta (Pépin *et al.*, 2020 ▸) were investigated up to a pressure of several Mbar using a 35 J portable laser, delivering for the first time high-quality data in both the XANES and the EXAFS range for materials under dynamic com­pression.

At the ESRF, dynamic com­pression was also coupled to X-ray Imaging (XRI) (Rutherford *et al.*, 2017 ▸; Olbinado *et al.*, 2018 ▸; Yanuka *et al.*, 2019 ▸; Miller *et al.*, 2019 ▸; Derrick *et al.*, 2019 ▸; Cohen *et al.*, 2019 ▸; Escauriza *et al.*, 2020 ▸; Farbaniec *et al.*, 2021 ▸) and X-ray Diffraction (XRD) (Briggs *et al.*, 2019 ▸; Pépin *et al.*, 2019 ▸) on the ID19 and ID09 beamlines, respectively. The success of these first experiments led to the launch of the High Power Laser Facility (HPLF-I) project, that foresees the coupling of a 100 J laser to the ID24 beamline to perform laser shock and ramp-com­pression experiments probed by time-resolved XAS.

Among other facilities of this kind, *i.e.* MEC at LCLS (Glenzer *et al.*, 2016 ▸), BL3-EH5 at SACLA (Inubushi *et al.*, 2020 ▸), DCS at APS (Wang *et al.*, 2019 ▸) and HED at EuXFEL (Descamps *et al.*, 2020 ▸), HPLF-I is the only one dedicated to and optimized for XAS in both the XANES and the EXAFS range. However, XANES of laser-shocked Fe has also been measured at MEC (Harmand *et al.*, 2015 ▸), but with limited data quality, mostly due to intrinsic intensity fluctuations of the X-ray beam, leading to the need for averaging over multiple acquisitions. High-quality single-pulse EXAFS, under laser-shock com­pression, has also been demonstrated recently at APS (Das *et al.*, 2020 ▸), but with insufficient energy resolution for the XANES region.

Extension to other X-ray techniques, such as XRI, XRD and XES (X-ray Emission Spectroscopy), is foreseen at HPLF in a second phase (HPLF-II after 2023). In this article, we present the first phase of HPLF-I where the front-end of the Amplitude laser, delivering 15 J, was coupled to beamline ID24, together with related technical details and initial experimental results.

## 2. Experimental setup

### 2.1. Energy-dispersive beamline ID24

The ED-XAS beamline ID24 (Pascarelli *et al.*, 2016 ▸) of the ESRF is optimized to couple the time-resolved ED-XAS technique under extreme conditions (pressure, temperature and magnetic field). Taking advantage of the dispersive scheme, dynamic com­pression is one of the main emerging applications (Torchio *et al.*, 2016 ▸; Mathon *et al.*, 2016 ▸).

A divergent X-ray beam is focused and energy dispersed by a bent crystal polychromator. The X-ray beam is focused on the target and the transmitted X-rays diverge onto a position-sensitive detector. As a result, the whole X-ray absorption spectrum is acquired at once, with no moving com­ponents during the acquisition. The XH detector (Borri *et al.*, 2021*a* ▸,*b* ▸), developed for pump–probe single-pulse meas­urements (see §2.2, *X-ray detector*), is capable of isolating a single X-ray bunch in different filling modes of the ESRF, namely, 4-bunch mode, 16-bunch mode and 7/8+1 mode (see Table 1 ▸). Therefore, the ultimate time resolution is given by the X-ray pulse length [100 ps FWHM (full width at half-maximum) at the ESRF] con­voluted with the temporal jitter between the drive laser and the X-ray bunch itself. The jitter is measured to be less than 50 ps RMS (root mean square) and the convolution with the X-ray bunch length gives 65 ps RMS (or 155 ps at FWHM). This is well suited to laser-shock-induced dynamic com­pression, as typical shock-time scales and most of the related processes are in the several hundreds of picosecond and nanosecond ranges.

ID24 provides X-rays in the energy range 5–28 keV using a combination of U27 and U32 undulators. So far, single-pulse measurements up to 20 keV have been performed under ambient conditions (Mathon *et al.*, 2016 ▸) and up to 11 keV under shock com­pression (*L*_3_-edge of Bi; see §4.3, *Bismuth*). Depending on the working energy and the beamline setup, the X-ray focal spot ranges from 3 to 50 µm FWHM horizontally and from 3 to 100 µm FWHM vertically. A small beam size is an important aspect in laser-shock experiments. Indeed, in order to probe a spatially homogeneous shock state, the X-ray beam must be much smaller than the laser spot and the shock front at the target position. A small X-ray spot will thus allow the use of smaller phase plates for the laser, increasing the available deposited power density and therefore extending the achievable pressure and temperature domains. However, when using a small phase plate, the target design, and especially the target thickness, has to be carefully considered to limit border effects and keep a homogeneous shock at the sample position (Remington *et al.*, 2006*a* ▸).

**Figure 1:**
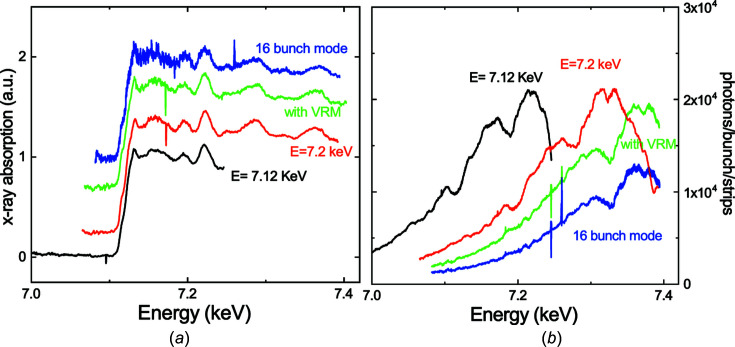
(*a*) Single-pulse XAS measurement of Fe (3.5 µm deposition on a diamond substrate) under ambient conditions in different configurations. Black, red and green lines are spectra acquired during the 4-bunch mode, while the blue spectrum was acquired in the 16-bunch mode. In the black spectrum, the spectrometer is aligned so that the Fe *K*-edge is in the middle of the energy range; in the red spectrum, the edge is moved towards low energies to increase the energy range; the green spectrum is the same as the red spectrum, but with the beam focused vertically using an additional mirror (VRM); and the blue spectrum is the same as the green spectrum but in the 16-bunch mode. (*b*) The corresponding direct beams (colours matching).

ID24 provides a 10^13^–10^14^ photons per second flux on the sample, depending on the energy (Pascarelli *et al.*, 2016 ▸), in multibunch mode (ring current 200 mA). At the Fe *K*-edge, this translates into an average of 1.5 × 10^4^ photons/bunch/strip in the 4-bunch mode (ring current 40 mA) (Mathon *et al.*, 2016 ▸), which is confirmed by our measurements after XH detector calibration *via* a calibrated Si diode. Fig. 1 ▸ reports the single-pulse reference measurements performed on Fe targets at beamline ID24 (L branch) in different configurations (left panel) and the corresponding direct beams (right panel). The photon distribution of the direct beam at the Fe *K*-edge is not flat but increases with energy. Therefore, different choices can be made in the beamline alignment to favour a higher signal-to-noise ratio (S/N) in the edge range (black spectrum) or a larger energy range at the expense of higher noise in the XANES (red spectrum). The average noise levels in the pre-edge range are 0.0056 and 0.0065 for the black and red spectra, respectively [same a.u. as in Fig. 1 ▸(*a*)]. For the black and red spectra, the beam was only focused horizontally by the polychromator (the beam size was around 5 µm × 90 µm, H × V FWHM).

Vertical focusing is achieved using an additional vertically refocusing mirror (VRM). This mirror cannot be placed under vacuum, but is fluxed with N_2_, which explains the slight increase of the average noise level to 0.012 in the pre-edge range (green spectrum).

Black, red and green spectra were acquired during the 4-bunch mode, the most favourable for single-pulse acquisition, as the current in one bunch reaches 10 mA and bunches are separated by more than 700 ns, allowing for a longer charge-collection time with the XH detector.

We show a single bunch acquisition during the 16-bunch mode again with VRM in place (blue spectrum). The current in a single bunch in this mode is around 5 mA and bunches are separated by only 176 ns, which accounts for the higher noise (0.16 on average in the pre-edge range).

Despite this, single-bunch spectra in 16-bunch mode are exploitable at least in one energy region at a time, *i.e.* by setting the beamline alignment to optimize XANES or EXAFS. Data quality for single-pulse spectra acquired in 7/8+1 mode is expected to be closer to that of the 4-bunch mode as the current in the single bunch reaches 8 mA. Single-pulse measurements in the 7/8+1 mode were performed on Fe alloy targets which were showing radiation damage and were thus protected by attenuating the beam with thick kapton foils (see §4.2, *Fe_*x*_O_*y*_, Fe–10 wt%Ni and Fe–3.5 wt%Si*). A direct com­parison of the S/N ratio is therefore not possible here.

The energy resolution of an energy-dispersive spectrometer depends on the central energy, *E*_0_, the crystal diffracting planes (*h*, *k* and *l*), and the geometry, *i.e.* the focusing distance and detector distance.

**Figure 2:**
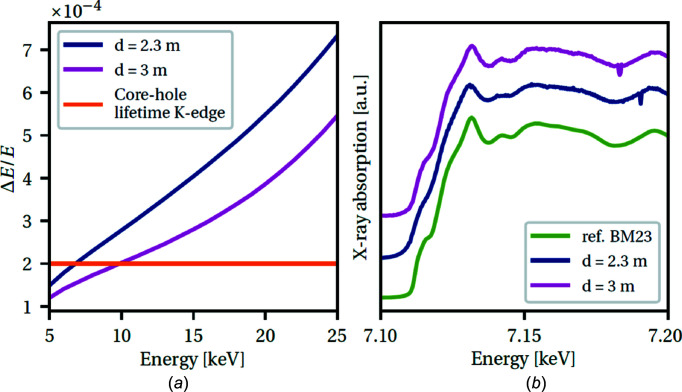
(*a*) Energy resolution as a function of energy for a Bragg polychromator using an Si(111) crystal focusing at a distance 1.1 m. The detector is placed at 2.3 m (blue curve) and 3 m (purple curve). (*b*) XANES spectra of Fe, where the blue and purple lines are spectra acquired on ID24 under the same conditions, and the green line is a reference spectrum from BM23.

The contributions to the effective angular spread are: (i) the spatial resolution of the detector, (ii) the Darwin width of the curved polychromator crystal, (iii) the size of the X-ray source and (iv) the spread of the monochromatic beam on the detector due to the Borrmann fan (only in the case of Laue geometry) (Pascarelli *et al.*, 2016 ▸). In the case of dynamic com­pression experiments, the focusing distance is 1.1 m and the XH detector can be placed at up to 3 m from the sample (4.1 m from the polychromator). In Bragg geometry, Fig. 2 ▸ (left panel) shows that our calculated energy resolution *dE*/*E* is better than the core hole lifetime broadening for the *K*-edges (*dE*/*E* < 2 × 10^−4^) up to *E* = 10 keV, and increases up to 5 × 10^−4^ at *E* = 25 keV. However, above 15 keV, the Laue geometry becomes preferable to avoid the effect of the asymmetrical reflectivity profile of the highly curved Bragg-type crystal, degrading energy resolution and creating an asymmetrical focal spot. In Fig. 2 ▸(*b*), we show two example spectra at the Fe *K*-edge, acquired in Bragg geometry with Si(111), with different sample-to-XH-detector distances in com­parison with a reference spectrum from a standard XAS beamline (BM23 at ESRF). The energy resolution improves at larger detector distances (purple curve). Depending on the scientific case, *i.e.* the XAS features that need to be resolved, one can choose to optimize the energy resolution or the flux (S/N ratio) by choosing a larger or smaller detector distance, respectively.

### 2.2. X-ray detector

One of the most critical elements in the setting up of single-bunch XAS measurements to probe laser-shocked matter has been the development of the XH detector. This detector is devoted to fast time-resolved applications, such as single-bunch pump/probe mode and film mode experiments. The critical part is linked to the Ge sensor technology, which is far less developed than Si technology. Si sensors are not usable due to strong radiation damage when exposed to the direct beam. The development of the Ge strip detector is the result of a long-standing collaboration between the ESRF and the Science & Technology Facility Council (STFC, Daresbury Laboratory, UK) (Borri *et al.*, 2021*a* ▸,*b* ▸). The acquisition electronics features a readout time of 2.8 µs, allowing one acquisition per ESRF ring revolution to be performed. The minimum integration time of around 100 ns allows the isolation of a single bunch in 4-bunch and 16-bunch modes, and the main intense bunch in the 7/8+1 mode (see Table 1 ▸). The XH detector has 1024 strips (rectangular pixels) of 25 µm width, 50 µm pitch and 5 mm height, leading to a total active area of 51.2 mm × 5 mm.

The original system was delivered to the ESRF and used successfully for laser shock dynamic com­pression experiments (Headspith *et al.*, 2007 ▸; Torchio *et al.*, 2016 ▸; Pépin *et al.*, 2020 ▸). Since 2017, an upgraded design was manufactured, with improved thermal performances and new germanium sensors from Mirion Technologies (Borri *et al.*, 2021*a* ▸,*b* ▸).

### 2.3. Laser

**Figure 3:**
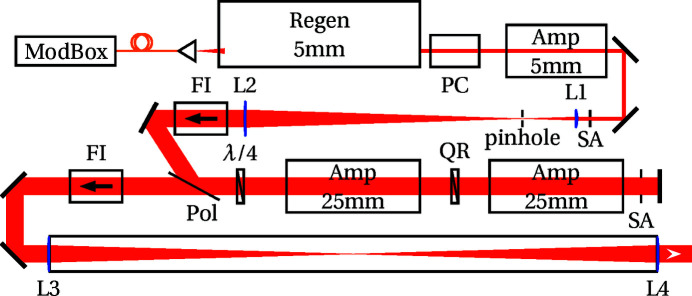
Optical layout of the laser front-end; see text for details. PC is the Pockels cell, Amp are the rod amplifiers with their diameter for indication, SA are the apodizing apertures, L are the lenses, FI are the Faraday Isolators, Pol is the thin-film polarizer and QR is the quartz rotator.

The laser is a custom-made Intrepid system from the Amplitude Laser Group (Continuum division in San Jose, CA, USA). It contains the generation of the laser pulses, spatial and temporal shaping, and amplification up to 15 J. Fig. 3 ▸ presents the optical layout of the laser used as the shock driver for the experiments reported here.

The seed source laser is inside a Front-End ModBox from iXBlue (Besançon, Fr). A Distributed FeedBack laser generates a continuous wave at 1053 nm. Within the same ModBox and using only fiber com­ponents, electro-optic modulators perform the temporal pulse shaping of this continuous wave. The temporal pulse shaping pre-com­pensates for the non­linear pulse distortion that occurs in the amplifier chain and allows nanosecond pulses to be delivered with the required duration and shape on target. Before leaving the ModBox and entering free-space propagation, another electro-optic mod­ulator spectrally broadens the pulses for the Smoothing by Spectral Dispersion (SSD) system. The energy per pulse reaches the nJ level at the end of this all-fibre section. The laser pulses are then coupled to free-space propagation and injected into a linear regenerative amplifier (Regen or RGA). There, a 5 mm diameter rod amplifies the energy up to the mJ range in a few passes. At the output of the RGA, a Pockels cell (PC) acts as an isolation stage, while also improving the temporal contrast of the single pulse. It is then further amplified in a single-pass 5 mm diameter rod, enlarged through an afocal telescope up to its final diameter of 23 mm. The final amplification is performed by two 25 mm diameter rod amplifiers in a double-pass scheme with birefringent com­pensation through a quartz rotator plate, isolated on both sides by large-diameter Faraday isolators. A final in-vacuum telescope relays the image plane close to the output port of the laser enclosure.

All amplifiers are pumped by flash lamps. In order to keep the bandwidth induced by the SSD system and to avoid FM-to-AM modulations (Rothenberg *et al.*, 1999 ▸; Hocquet *et al.*, 2008 ▸), all amplifiers use Nd:glass rods. Given the limited thermal conductivity of the laser glass and the significant thermal load due to flash pumping, the repetition rate of the laser is limited to 0.1 Hz. To keep the thermal load as constant as possible, all the flash lamps are always triggered at 0.1 Hz and the overall alignment of the laser takes into account the induced thermal lensing. For easier alignment, it is possible to drive both 5 mm diameter amplifiers at 0.5 Hz without sig­nificant changes in alignment. To prevent maximum am­pli­fication during the alignment process while keeping the thermal lensing of the 25 mm diameter amplifiers constant, their flash lamps, still running at 0.1 Hz, can be time delayed by a few milliseconds. We also used this capability to define four different delays for the 25 mm diameter amplifier flash-lamp trigger resulting in four different energy output levels of 15, 10, 5 and 0.08 J, with the last corresponding to no amplification at all using a delay significantly larger than the fluorescence lifetime of the Nd:glass rods. These energy levels are selectable from the beamline control interface.

### 2.4. Synchronization

**Figure 4:**
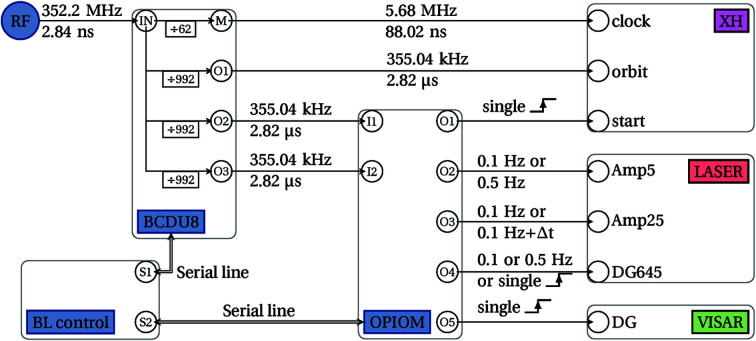
Schematic view of the synchronization system. The synchrotron RF is distributed to all instruments through a set of two cascaded in-house electronics (BCDU8 and OPIOM). The frequency and corresponding period are shown for each clock, while only the repetition rate is shown for trigger events.

The synchronization of all instruments was achieved through ESRF-developed electronics, namely, the Bunch Clock Delay Unit (BCDU8) and Octal Programmable I/O Module (OPIOM) cards (Fig. 4 ▸).

The BCDU8 takes as its only input the radiofrequency clock of the storage ring (RF master clock) and outputs synchronous pulses of adjustable delays and widths. Two of its output channels are capable of fine delays (11 ps steps). One of these channels is used for the laser timings. While providing fine delays on two channels, the total delays are limited to approximately half of the output period (*i.e.* ≈1.4 µs in our case). As our laser and detector require longer delays, the BCDU8 outputs are then fed to the OPIOM card.

This second electronic device pro­vides combinational logic between its inputs through a programmable logic device (PLD) and a microcontroller. The OPIOM works using its inputs as its time base, both for delays and pulse dur­ations. In our case, we have two input clocks running at 355 kHz (*i.e.* 2.82 µs ticks, corresponding to the ESRF storage ring revolution or bunch orbit). According to a precom­piled firmware, the OPIOM then generates the following output signals, each with its own coarse delay, repetition rate and duration: a single pulse trigger for the XH detector; a clock for the 5 mm amplifiers either at 0.1 or 0.5 Hz, with a fixed delay of 520 µs; a clock for the 25 mm amplifiers always at 0.1 Hz, with a default delay of 0 µs which can be increased by nearly 1.5 ms to prevent amplification while keeping the thermal load constant; a trigger for the internal delay generator of the laser driving the ModBox and the Pockels cells; a trigger for the shocks diagnostics. The OPIOM outputs drive the subsequent devices: the laser, and both X-rays and visible diagnostics. The overall timing scheme is depicted in Fig. 4 ▸, which shows the signal distribution from the synchrotron RF to the final instruments. As the synchronization is partly based on the bunch clock (RF/992), all acquisitions are performed following always the same single bunch within the storage ring. The synchronization setup then remains identical for all filling modes of the storage ring (see Table 1 ▸ for details), apart for the fine delay to superimpose the laser on the X-ray pulse.

This setup allows a com­plete synchronization of the facility based on the storage ring radiofrequency and also provides control for both the operating mode of the laser and the detector from the beamline control session.

Laser pulse/X-ray pulse fine synchronization is observed through a single ultrafast avalanche photodiode (APD) placed at the sample position. The laser delay is then adjusted with the fine delay output of the BCDU8 card in order to superimpose the rising edges of both X-rays and laser pulses.

The final High Power Laser Facility will benefit from a modern in-house-developed delay generator using the recently deployed RF over a White Rabbit network of the ESRF (Goujon *et al.*, 2018 ▸).

### 2.5. Target chamber

**Figure 5:**
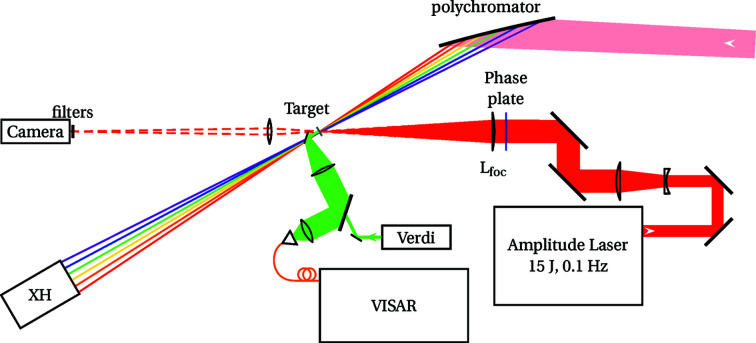
The configuration of the beams (not to scale). The beamline pink beam is diffracted and focused normal to the sample surface (pink then sketched as a few rainbow rays). The laser, in red, arrives at 30°. Downstream is the X-ray detector. A flip mirror can be inserted in the X-ray beam path for VISAR analysis (green). A direct line-of-sight camera can be used to record the laser focal spot in the absence of samples (dashed red). The vacuum vessel, its windows and the two in-vacuum microscopes for sample observation are not shown for clarity.

The interaction geometry was kept similar to previous experiments (Torchio *et al.*, 2016 ▸; Pépin *et al.*, 2020 ▸). The X-rays are normal to the sample surface, while the drive laser arrives at 30°. Fig. 5 ▸ details the beam configuration.

Two in-vacuum microscopes offer sample visualization from upstream and downstream. In order to observe perpendicular to the sample surface, a flip mirror folds the beam path for each microscope. These flip mirrors are then retracted to free the X-ray beam path.

A Point VISAR (Velocity Interferometer System for Any Reflector) – model Valyn VISAR with a Verdi CW laser from Coherent – was used to characterize the loading conditions using aluminium samples. Given the interaction geometry, as depicted in Fig. 5 ▸, the X-rays and the VISAR cannot be operated at the same time. Hence, the VISAR was used offline by inserting a small mirror close to the target rear face. This is a clear limitation of the current interaction geometry. In order to perform XAS and loading conditions measurements simultaneously, the future interaction geometry for HPLF will be modified. The same relative angle between the X-rays and laser drive will be kept but the sample normal will sit in between both beams (*i.e.* X-rays incident at +15° and laser drive at −15°). This opens up the rear side enough to install a normal-incidence optical access for visible diagnostics of the shock conditions. A line VISAR and a SOP (Streaked Optical Pirometry) system will be implemented in HPLF.

The vessel was evacuated down to 10^−4^ mbar with a turbomolecular pump backed up by a dry pump. Additional under-vacuum tubes were used between the polychromator and the target chamber and in front of the detector in order to minimize X-ray absorption in air.

**Figure 6:**
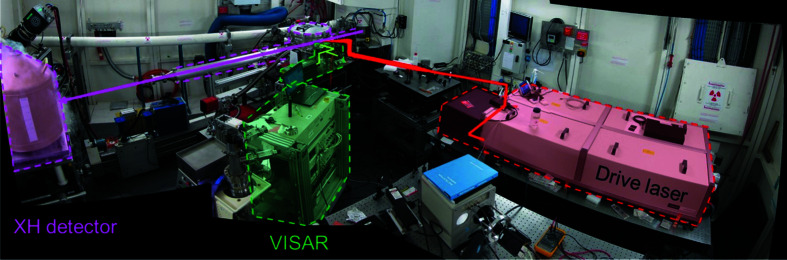
A photograph of the experimental hutch in November 2018. Beams are shown as solid arrows, whereas instruments are shown as dashed lines. X-rays are shown in pink, the drive laser in red and the shock diagnostics in green. For scale, the laser is 2 m long.

Fig. 6 ▸ provides an overview of the experimental hutch during the experiments.

## 3. Laser front-end commissioning

### 3.1. Laser characterization

The laser breadboard (2 m × 1 m) was installed inside the experimental hutch. All the electronics, power supplies and cooling group were installed inside the control cabin providing easy access.

**Figure 7:**
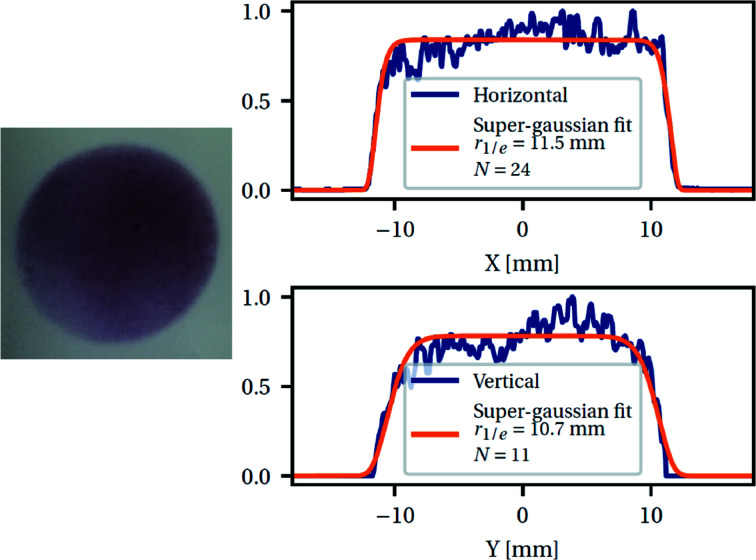
A photograph of the beam on burnpaper at the laser output (not focused and without a phase plate, left). Horizontal and vertical profiles recorded with a beamviewer (Gentec Beamage) and the corresponding super-Gaussian fits (right) with profile 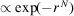 .

The laser beam cross section at 15 J is shown in Fig. 7 ▸.

Beam inhomogeneities are limited to, respectively, 8 and 10% RMS over the central parts (defined as 90% of the diameter) of the horizontal and vertical profiles, and there are no sharp spots. The cross section of the beam is only slightly elliptical (minor/major = 93%) and is missing small amounts of energy in the lower part, as can be seen in the unbalanced vertical profile. The edges are sharp (super-Gaussian order >10 along both axes).

Repeatability of the laser parameters (delivered energy, temporal shape and beam pointing) is of paramount importance to be able to duplicate shots or com­pare successive shots with different time delays with respect to the X-ray probe. Hence, the qualification runs were performed over 50 shots at a default repetition rate of 1 shot every 4 min (maximum shot rate for the future 100 J laser).

The energy output was 14.5 J on average with an energy stability of 1.2% RMS (8.8% peak-to-peak).

The beam-pointing stability was measured with a 1 m focal length lens at 14 µrad RMS (45 µrad peak-to-peak horizontally and 34 µrad peak-to-peak vertically).

**Figure 8:**
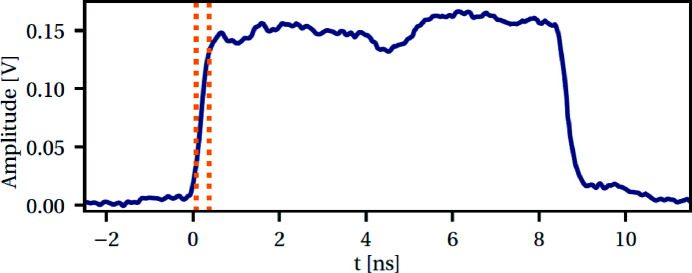
Laser temporal profile recorded with a fast photodiode (EOT ET-3500) connected to a fast oscilloscope (LeCroy WaveMaster 20 GHz). Orange dotted lines represent the rise time between 20 and 80% of the maximum, *i.e.* 300 ps.

In 2018, the temporal pulse shape was fixed to a 9 ns square, with a rise time of 300 ps (20–80%) and a contrast better than 10^4^ (detection limit with the present setup) on the nanosecond timescale before the main pulse, as shown in Fig. 8 ▸. This pulse shape can be improved, especially with respect to the rise time between 10 and 90% (1.4 ns), and the plateau fluctuations. Concerning reproducibility, the qualification tests (50 shots) showed that the amplitude of the plateau was reproducible within ±10% peak-to-peak and the width was stable at 8.7 ns (40 ps RMS).

The future facility will offer square pulses from 2 to 15 ns and also provide the possibility of pulse shaping for ramp-com­pression experiments.

### 3.2. Laser beam transport, focusing and alignment

**Figure 9:**
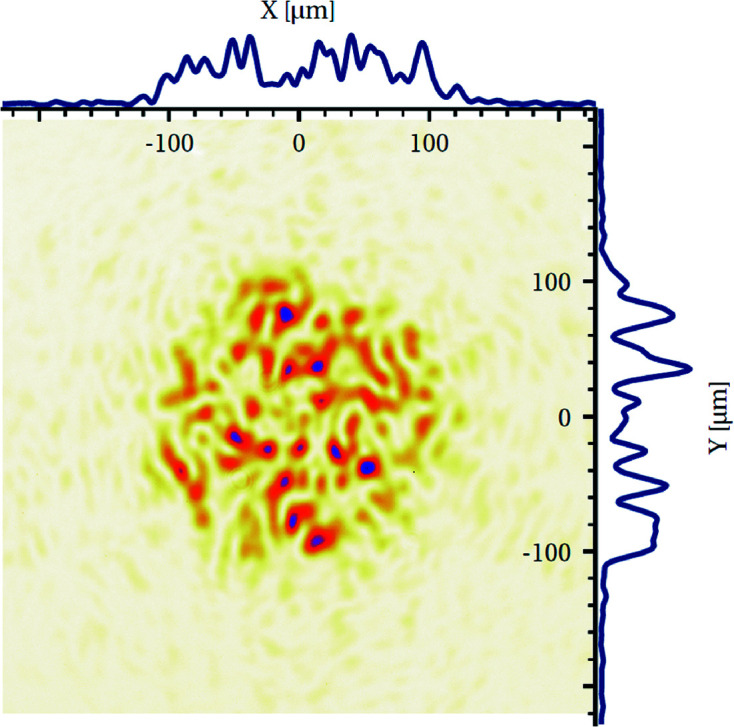
Capture of the laser spot at the sample position using a hybrid phase plate designed to generate a 250 µm focal spot. The profiles shown at the top and to the right are 40 µm thick lineouts through the focal spot at *Y* = 0 and *X* = 0, respectively. Full width at half-maximum (FWHM) is close to 200 µm along both axes. Enclosed energy measurements on this figure show 50% of the energy inside a 180 µm diameter, 70% inside a 226 µm diameter and 80% inside a 290 µm diameter.

From the laser output port, a set of mirrors steers the laser beam up to the interaction chamber. In order to match the size of the phase plates used in previous experiments (Torchio *et al.*, 2016 ▸), the beam diameter at the laser front-end output is enlarged by a Keplerian telescope from 23 to 40 mm approximately midway between the laser output port and the sample (for a total of about 4 m of free propagation). The focusing lens (*f* = 350 mm) is placed before the laser input port of the vacuum chamber, the phase plate being in between the focusing lens and the vacuum window. An imaging system (microscope objective and a camera) is placed on the axis of the laser beam and monitors the focal spot shape and the size at the target position at low energy. Fig. 9 ▸ shows a typical focal spot when using a 250 µm Hybrid Phase Plate. The obtained profile is quite inhomogeneous, with intensity variation up to 50% over the micrometric scale. However, recent studies at LCLS (Smith, 2016 ▸) have shown that in laser-driven dynamic com­pression experiments, the laser spatial profile inhomogeneity can be smoothed by a sufficiently thick target ablator thanks to thermal conductivity. Moreover, the future facility will feature a larger input beam size which will increase the density of smaller speckles. It will also feature Smoothing by Spectral Dispersion (SSD) which will smear out the speckles along one axis.

The X-ray focal spot is materialized by scanning a 50 µm pinhole in the X-ray beam and visualizing the pinhole position on the on-axis image. The laser focal spot of Fig. 9 ▸ is then moved to the same position using the translations of its focusing lens. Fine spatial overlap is finally checked through direct observation of low-energy laser shots on plastic samples placed at the sample position.

The laser parameters and overall alignments were found to be reproducible and stable over several days, with some minor adjustments to the regenerative cavity due to the temperature increase inside the hutch and fine alignments on the target. This allowed the successful collection of XAS spectra and the summation of successive identical shots as described in §4.3 (*Bismuth*).

## 4. First results on Fe alloys, Fe oxides and Bi

### 4.1. Targets

**Figure 10:**
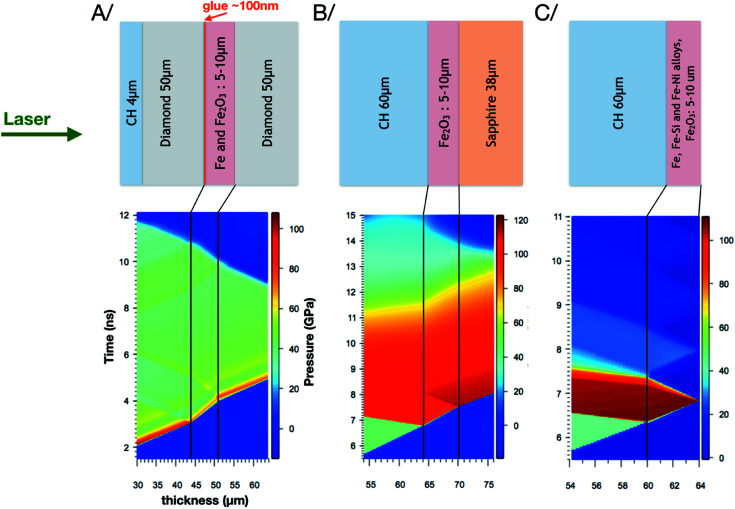
Fe, Fe alloys and Fe_2_O_3_ target designs, and the corresponding hydrodynamics simulations for the case of Fe_2_O_3_.

The iron, iron alloy and iron oxide targets were designed, fabricated and assembled at the IMPMC laboratory. Three major target designs were used to ensure both homogeneous and well-controlled thermodynamic conditions at the time of the X-ray probe, a few ns after the laser impinges on the target front face. They are shown in Fig. 10 ▸.

The first design, labelled *A* in Fig. 10 ▸, was used in a previous ID24 laser shock experiment (Torchio *et al.*, 2016 ▸). It consists in a 4 µm plastic parylene-N ablator, a 40 µm diamond and a deposited Fe or Fe_2_O_3_ layer on a second 40 µm diamond.

The shock Hugoniot conditions were determined by hydro­dynamic simulations with the *MULTI* code (Ramis *et al.*, 1988 ▸) using the SESAME EOS Table 2150 for Fe, 7440 for Fe_2_O_3_, 7411 for sapphire and 7592 for CH (initial density of 1.044 g cm^−2^). The input laser pulse is a square pulse of 9 ns with an intensity of 1.8 × 10^12^ W cm^−2^. This laser intensity was calibrated using CH-Al targets with the same ablator thickness, by adjusting the simulated free surface velocity and shock arrival time to those obtained from off-line VISAR measurements. Examples of simulations are shown in Fig. 10 ▸. Unfortunately, it has not been possible to perform VISAR measurements of the targets directly because of radiation damage from the continuous VERDI laser.

For target design *A*, the simulations show that the thermodynamic conditions remain stable in the range 7–8 ns and correspond to a pressure of about 40–50 GPa for both Fe and the alloys. The two other target designs, labelled *B* and *C* in Fig. 10 ▸, allow us to reach a higher pressure using the impedance mismatching between the plastic ablator and the investigated samples.

The *B* target design consists of a multilayer of a deposited parylene-N (60 µm) and a deposited Fe_2_O_3_ (5.9 µm) on a 40 µm sapphire window. This design allows us to keep the thermodynamic conditions in the range 2–3 ns thanks to the similar impedance between our samples and the sapphire window. The *C* target design corresponds to a 60 µm parylene-N, that was either directly deposited on Fe–Si and Fe–Ni alloys or used as a substrate for Fe and Fe_2_O_3_ depositions. For this target design, the thermodynamic conditions release rapidly due to the fast expansion of the rear side of the target. For both the *B* and *C* designs, the achieved pressures under the conditions of the experiment are around 110–120 GPa (for both Fe and alloys), which go far beyond the bcc–hcp phase transitions of Fe and Fe alloys and beyond the Mott phase transition of Fe_2_O_3_ at about 50 GPa (Sanson *et al.*, 2016 ▸). Polycrys­tal­line Fe, Fe–Si and Fe_2_O_3_ samples were obtained by physical vapour de­pos­ition at IMPMC and their thicknesses were optimized to enhance the X-ray absorption signal. The Fe–Ni alloys were polycrystalline commercial foils from HMW Hauner GmbH & Co. KG.

The Bi targets were a multilayer made of a sapphire window (200 µm thickness) glued on to 10 µm thick Bi foils from Goodfellow which are provided on a support layer of polyimide on the back side (125 µm). This target design allows us to work in a con­fined plasma-driven shock scheme, where the laser ablation does not occur at the target front face, but at the target/sapphire interface (Fabbro *et al.*, 1990 ▸). This scheme provides a reproducible pressure time evolution in the Bi sample, allowing the shock states to be maintained for several nanoseconds, but reaching limited pressures (5–10 GPa). More details about the expected conditions for the results shown here are reported in §4.3 (*Bismuth*).

### 4.2. Fe_*x*_O_*y*_, Fe–10 wt%Ni and Fe–3.5 wt%Si

The investigation of Fe-rich alloys, such as FeNi, FeSi and Fe oxides, under extreme conditions of pressure and temperature is of crucial interest for the understanding of the com­position and evolution of the mantle and core of the Earth and Super-Earth exoplanets (Wicks *et al.*, 2018 ▸; Boulard *et al.*, 2019 ▸; Miozzi *et al.*, 2020 ▸; Torchio *et al.*, 2020 ▸; Boccato *et al.*, 2020 ▸). The influence of lighter or minor elements on the Fe phase diagram is still not fully understood.

**Figure 11:**
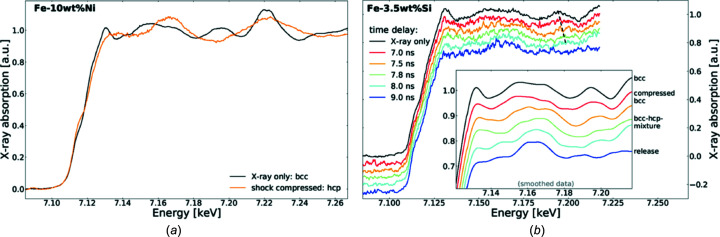
Normalized single-shot XAS spectra of (*a*) Fe–10 wt%Ni (4-bunch mode) and (*b*) Fe–3.5 wt%Si (7/8+1 bunch mode) recorded at various time delays between the optical laser and X-ray pulses. The spectra under ambient conditions, representing the bcc structure, are shown in black.

In the case of Fe–10 wt%Ni, we observe an unambiguous laser-induced bcc-to-hcp transition, similar to pure Fe (Torchio *et al.*, 2016 ▸) [Fig. 11 ▸(*a*)]. On the contrary, mixed bcc–hcp phases are observed in Fe–Si alloys: the bcc feature at around 7.195 keV (indicated with dashed lines in the figure) is visible in the normalized XAS spectra up to an 8 ns time delay between the optical laser and the X-ray pulses [Fig. 11 ▸(*b*)]. The addition of a small percentage of Si (3.5 wt%) to Fe thus seems to kinetically hinder the com­plete bcc–hcp phase transformation similar to recent X-ray diffraction measurements under laser com­pression that have shown no hcp phase transitions for Fe–16 wt%Si (Fischer & Campbell, 2015 ▸; Wicks *et al.*, 2018 ▸; Edmund *et al.*, 2019 ▸; Krygier *et al.*, 2021 ▸). It should be noted that the S/N ratio in the FeSi alloy data is lower than in the FeNi alloy. During the alignment, the sample was showing X-ray beam damage due to the high flux of the 7/8+1 mode, and was therefore protected by four kapton foils of 75 µm, thus reducing the photon flux by 30%. The same problem occurred for Fe_2_O_3_ described below. In the future HPLF, the installation of motorized attenuation will be implemented to protect the sample during alignment and only expose it during the shot.

**Figure 12:**
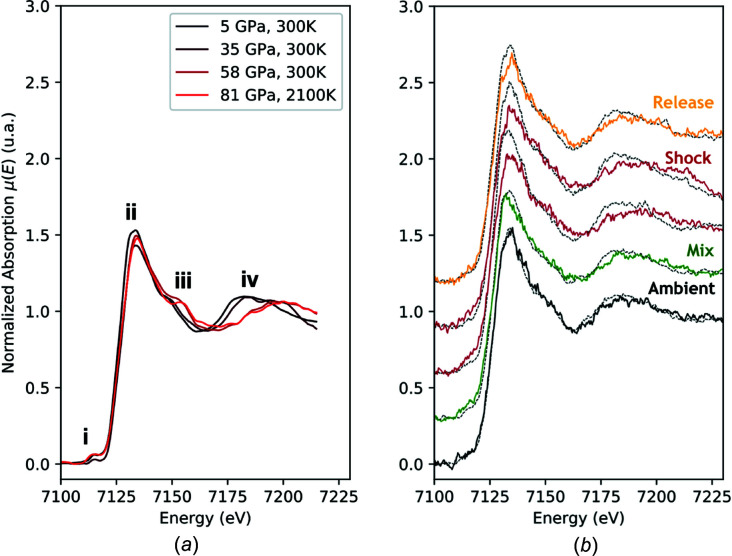
(*a*) X-ray absorption spectra of Fe_2_O_3_ at pressures of 5, 35 and 59 GPa and ambient temperatures, and at 81 GPa and 2100 K, using the LH-DAC apparatus at ESRF ID-24 (Boulard *et al.*, 2019 ▸). (*b*) X-ray absorption spectra of Fe_2_O_3_ under shock at an estimated pressure and temperature of 120 GPa and 1500 K, and under adiabatic release at ambient pressure and 1000 K (SESAME EOS 7440). The black dotted spectrum is the ambient reference.

In the case of Fe_2_O_3_, we show XANES measurements under shock and release conditions in Fig. 12 ▸(*b*). Those data were taken with target design *B* which includes a sapphire window for maintaining the pressure and temperature conditions for several ns and that allows pressures of ∼120 GPa to be reached thanks to the impedance mismatching between the plastic ablator and the Fe_2_O_3_ deposited layer.

In Fig. 12 ▸(*a*), we show Laser-Heated Diamond-Anvil-Cell (LH-DAC) data obtained at 80 GPa and 1500–2000 K for com­parison (Boulard *et al.*, 2019 ▸). In the LH-DAC data, we observe (i) a change of the pre-edge feature, (ii) a decrease of the absorption signal, (iii) a slight modification of the XANES shoulder at 7150 eV and (iv) a shift of the first XANES oscillation toward high energies. This behaviour was also observed in the recent article by Sanson *et al.* (2016 ▸) and was attributed to the High Spin–Low Spin (HS-LS) phase transition at ∼53 GPa and was followed by the formation of the orthorhombic structure with the space group *Aba*2 (Bykova *et al.*, 2016 ▸). Similar observations were made in the Fe_2_O_3_ shock data obtained during this experimental campaign [see Fig. 12 ▸(*b*)]. The feature labelled ‘iv’ shifts to higher energies, indicating the com­pression of the lattice parameters while the intensity of feature ‘ii’ decreases and a slight modification of pre-edge ‘i’ is observed. This indicates that the HS-LS transition was crossed and that most probably the orthorhombic *Aba*2 structure of Fe_2_O_3_ was formed within the duration of our experiment, *i.e.* less than 1 ns after the arrival of the shock in the Fe_2_O_3_ sample.

Further studies under optimized experimental conditions and at a higher laser energy would allow higher pressure ranges to be reached and high-pressure phases and the electronic local order of Fe_2_O_3_ to be disentangled. It should be noted that the increased resolution in the pre-edge range of the static data is mainly due to the use of the Si(311) Bragg polychromator.

### 4.3. Bismuth

**Figure 13:**
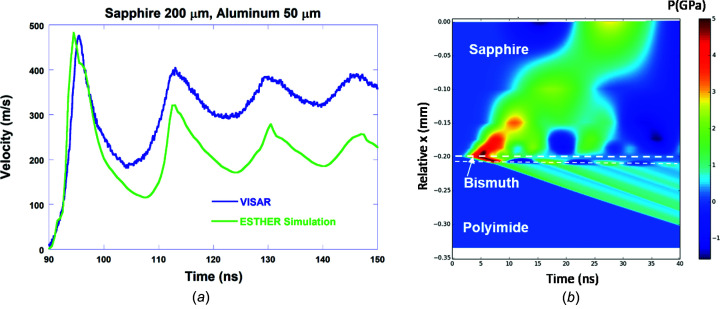
(*a*) Com­parison of the VISAR measurement and the ESTHER simulation of a target made of a window of 200 µm of sapphire and 50 µm of Al. (*b*) The pressure map of a Bi target (sapphire/Bi/polyimide) when the same simulation parameters are applied. The used EOS in the simulations are: SESAME 3720 for Al, SESAME 7411 for sapphire and BLF for Bi.

Bismuth is a prototype example of a material whose PT phase diagram changes as a function of the strain rate. Despite recent advances on its phase diagram under dynamic solicitation (Gorman *et al.*, 2018 ▸; Pépin *et al.*, 2019 ▸), questions remain about high-pressure phase stability as a function of kinetics (Fig. 13 ▸). Here we investigate Bi under laser-induced dynamic com­pression probed by time-resolved XAS, in particular looking for the signature of the Bi^I^ (rhombohedral) to Bi^V^ (body-centred-cubic) transition that occurs around 8 GPa in static com­pression and already from 4 GPa in dynamic com­pression (Chen *et al.*, 2016 ▸; Pépin *et al.*, 2019 ▸).

**Figure 14:**
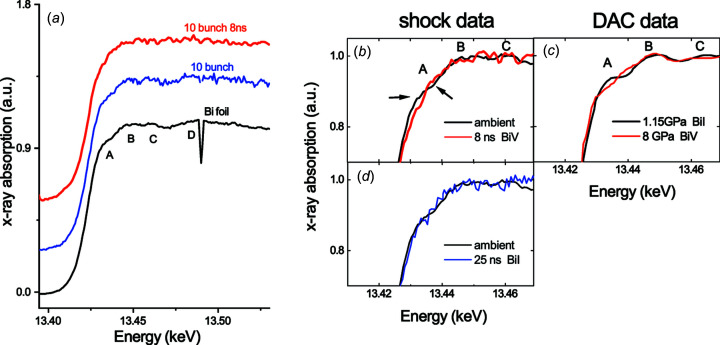
(*a*) Bi foil reference spectrum (black), Bi target spectrum obtained summing 10 X-ray pulses (blue) and shocked Bi target at 8 ns of laser delay (average of 10 X-ray pulses, red). (*b*) Observation of the Bi^I^ to Bi^V^ transition at 8 ns and (*c*) com­parison with literature static data. (*d*) Recovery of the Bi^I^ phase at 25 ns (blue spectrum, average of 5 X-ray pulses).

The laser was focused down to 500 µm at the sample position. Single-bunch XAS measurements were performed at the Bi *L*_3_-edge (13.4 keV) using the XH detector with the 7/8+1 filling mode. XANES features of the Bi *L*_3_ absorption edge are quite small [**A**, **B**, **C** and **D** in Fig. 14(*a*) ▸]; the sum of 8–10 single X-ray bunch acquisitions was needed to discern them.

Compression conditions were estimated by hydrodynamic simulations using the ESTHER code (Colombier *et al.*, 2005 ▸). However, because of the polyimide backing (Bi foils from Goodfellow are not stand alone), measurements could not be performed directly on the Bi targets. Therefore, the simulation parameters were preliminarily calibrated by com­parison with VISAR measurements on Al targets with the same front sapphire window, following a procedure previously used for Bi (Pépin *et al.*, 2019 ▸) (see Fig. 13 ▸). According to hydrodynamic simulations, at 8 ns Bi is in a uniform pressure state of around 5 GPa, and a full conversion into Bi^V^ is expected.

The pressure-induced Bi^I^ to Bi^V^ transition shows up in tiny changes in the XANES region, as is visible from static com­pression (DAC) data in Fig. 14 ▸(*c*) (Chen *et al.*, 2016 ▸).

Our XANES spectrum obtained under dynamic com­pression shows changes at 13.432 and 13.437 keV [indicated by arrows in Fig. 14 ▸(*b*)], similar to that obtained in static com­pression, and is thus com­patible with the observation of the transition. Starting from 13.45 keV, the shocked spectrum shows a flat shape as a consequence of thermal disorder [red spectrum in Fig. 14 ▸(*a*)]. At 25 ns, the Bi state reverts to ambient-pressure conditions. The spectrum acquired at 25 ns is noisier since it was only averaged over five accumulations. However, its edge shape looks like that of the ambient reference, which is com­patible with the recovery of the Bi^I^ structure. The detection and phase diagram location of the other expected phases (Bi, Bi^II^ and Bi^III^) might be the subject of future work.

## 5. Conclusions and perspectives

We presented here the commissioning results of the laser front-end of the future High Power Laser Facility at ESRF. The laser was characterized and coupled to the dispersive XAS beamline ID24. First-user experiments were performed and new X-ray Absorption data of iron com­pounds and bismuth were collected under laser-induced dynamic com­pression. Several aspects of the preliminary setup described here will be improved for the final version of HPLF. The final interaction geometry will allow simultaneous XAS and loading condition measurements (both line VISAR and SOP), minimizing the number of targets needed and improving the reliability of the pressure/temperature conditions associated with the XAS spectra, not having to rely on the reproducibility of the target and laser conditions.

The synchronization scheme will also be refined to be more versatile and easier to operate.

During the ESRF shut down for the major Extremely Brilliant Source (EBS) upgrade of the storage ring (2019), the laser was shipped back to Amplitude Technologies for further improvements and to build the amplification stage up to 100 J. The new laser was installed at the ESRF in a new dedicated clean room in July 2021. The High Power Laser Facility is scheduled to open for user experiments in December 2021 and will benefit from the new EBS of the ESRF. Thanks to the source and beamline upgrades, an increase of flux by a factor 2–5 (depending on the energy) is expected. In the longer term, the HPLF laser is planned to be shared with a second adjacent beamline to implement further time-resolved X-rays probes, such as X-ray diffraction, X-ray imaging and X-ray emission, for laser shock and ramp experiments.

## Figures and Tables

**Table 1:** ESRF filling modes and timings during HPLF-2018 runs

Mode	Pattern (current *versus* time)	SR current (mA)	Main bunch current (mA)	Δ (ns)
7/8+1	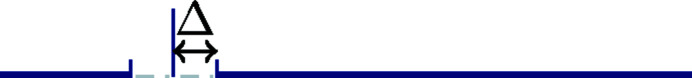	200	8	176
16-bunch	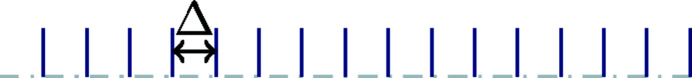	90	5.625	176
4-bunch	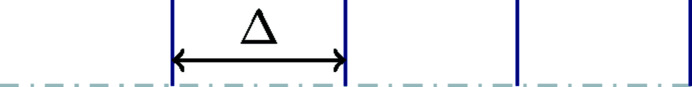	40	10	704
